# Flow reduction of a high‐flow arteriovenous fistula in a hemodialysis patient reveals changes in natriuretic and renin–angiotensin system hormones of relevance for kidney function

**DOI:** 10.14814/phy2.14989

**Published:** 2021-10-04

**Authors:** Christine L. Meyer‐Olesen, Kristine Lindhard, Niklas R. Jørgensen, Jens P. Goetze, Tobias Bomholt, Boye L. Jensen, Ditte Hansen

**Affiliations:** ^1^ Department of Nephrology Copenhagen University Hospital ‐ Herlev and Gentofte Copenhagen Denmark; ^2^ Department of Clinical Biochemistry Copenhagen University Hospital ‐ Rigshospitalet Copenhagen Denmark; ^3^ Department of Nephrology Copenhagen University Hospital ‐ Rigshospitalet Copenhagen Denmark; ^4^ Department of Cardiovascular and Renal Research University of Southern Denmark Denmark; ^5^ Department of Clinical Medicine University of Copenhagen Copenhagen Denmark

## Abstract

Arteriovenous fistulas (AVFs) are iatrogenic vascular connections established to allow high‐flow intravascular access for patients with chronic kidney disease requiring hemodialysis. The left‐right flow shunt results in changes in extracellular fluid volume and blood pressure‐controlling hormones that could affect the residual kidney function. We present a case where a female patient with a brachiocephalic AVF had a fistula flow of >4 L/min. To reduce the flow, a banding procedure was performed. The patient was examined prior to banding and 1 and 2 weeks thereafter. Banding resulted in a marked decrease in AVF flow from >4 to 1 L/min and was associated with reductions in N‐terminal pro‐brain natriuretic peptide of 51% and 67% at 1‐ and 2‐weeks post‐banding, respectively. Mid‐regional pro‐atrial natriuretic peptide concentrations were reduced post‐banding by 17% after 1 week and 25% after 2 weeks. After 1 week, renin, angiotensin II, and aldosterone levels in plasma decreased transiently by 44%, 47%, and >86%, respectively, and returned to pre‐banding levels after 2 weeks. Creatinine clearance tended to decrease while blood pressure and total body water increased 2 weeks after banding. This indicates that high‐flow AVF is associated with increased natriuretic peptides and hormones of the renin–angiotensin–aldosterone system, that may balance each other regarding fluid retention and hypertension and support remaining kidney function.

## Graphical abstract

A patient with end‐stage kidney disease and an arteriovenous fistula with extreme access flow of >4 L/min underwent flow reduction to 1 L/min by banding. Flow reduction was associated with increased total body water and blood pressure and reduction in natriuretic peptide hormones along with temporary decreased hormones of the renin—angiotensin—aldosterone system and a small decrease in creatinine clearance. This indicates that a high‐flow arteriovenous fistula is associated with hormonal changes that may balance each other regarding fluid retention and hypertension and support remaining kidney function.

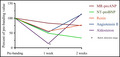

## 1 INTRODUCTION

Arteriovenous fistulas (AVFs) are the preferred vascular access for chronic hemodialysis in end‐stage kidney disease (Lok et al., 2020). Surgical creation of an AVF leads to a blood flow shunt from the arterial to the venous system. This significantly alters cardiovascular hemodynamics by decreasing the total peripheral vascular resistance and increasing the venous return to the heart thereby increasing the stroke volume and cardiac output (Duque et al., 2015; Guyton & Sagawa, 1961; Ori et al., 1996). Previous studies have shown an association between AVF creation and increased plasma concentrations of brain natriuretic peptide (BNP) and atrial natriuretic peptide (ANP) (Iwashima et al., 2002; Malík et al., 2009). Both hormones are secreted from cardiomyocytes stimulated by dilatation of the heart following volume load or cardiac failure (LaPointe, 2005; Yasue et al., 1994). The hormones share biological effects via the common natriuretic peptide receptor A (NPR‐A), located in vascular, kidney, and adipose tissue (Nishikimi et al., 2006; Sarzani et al., 1996). Among these effects are decreased blood pressure through direct vasodilatation, inhibition of catecholamine release, and inhibition of the renin–angiotensin–aldosterone system (RAAS) (Brunner‐La Rocca et al., 2001; Nishikimi et al., 2006). Furthermore, natriuretic peptides induce dilatation of the renal afferent arteriole and contraction of the efferent arteriole, thus increasing the glomerular filtration rate and natriuresis (Endlich et al., 1997; Epstein et al., 1998; Jensen et al., 1999).

Data from observational association studies raised the hypothesis that AVF establishment reduces blood pressure and slows kidney function decline in patients with end‐stage kidney disease and thus delays the time for hemodialysis initiation (Bénard et al., 2019; Dupuis et al., 2021; Golper et al., 2015; Sumida et al., 2016). The mechanism of this effect is not known. We speculated that the changes in BNP and ANP associated with AVF flow could directly affect glomerular filtration rate, RAAS, and the sympathetic nervous system. We present a case report, where a patient with end‐stage kidney disease and an AVF with extreme access flow of >4 L/min underwent flow reduction by AVF banding. To examine the hormonal changes and potential effect on blood pressure and kidney function associated with flow changes, we measured the plasma concentrations of NT‐proBNP (a proxy measure of BNP), MR‐proANP (a proxy measure of ANP), renin, angiotensin II, aldosterone, together with creatinine clearance, blood pressure, and body fluid composition before and after banding.

## 2 CASE REPORT

At a routine examination in June 2019, a hemodialysis patient presented with an AVF access flow of >4 L/min. The patient was a 52‐year‐old woman with hypertension and end‐stage kidney disease secondary to bilateral hydronephrosis of unknown cause. She began hemodialysis in November 2018 and received maintenance hemodialysis three times per week. The hemodialysis was performed without ultrafiltration as she maintained an adequate diuresis. Her vascular access was a left‐arm brachiocephalic AVF established in September 2017. In January 2019, she presented with a stenosis of the arteriovenous anastomosis causing low access flow of 0.17 L/min. A percutaneous transluminal angioplasty was performed resulting in an increase of access flow reaching >4 L/min in June 2019. The patient was asymptomatic, and a transthoracic echocardiography revealed a normal left ventricular ejection fraction of 55% and no diastolic dysfunction. Therefore, it was decided not to reduce the AVF flow at this point. By March 2020 the patient developed paresthesia of the left hand, which was interpreted as “steal” symptoms. Repeated measures revealed persistent high access flow ranging from 3.3 to >4 L/min, and a decision was made on flow‐reducing surgery by banding that was performed in June 2020. Three weeks after the operation the access flow was 1 L/min and symptoms of steal syndrome disappeared. During the study period, the patient received hemodialysis at home without ultrafiltration 4 h thrice weekly with a blood flow rate of 400 ml/min. The patient received the following medication: Furosemide, metoprolol, darbepoetin alfa, sevelamer, calcium carbonate, alfacalcidol, cholecalciferol, ergocalciferol, folic acid, B vitamins, and vitamin C. There were no changes in medication within the study period.

## 3 METHODS

Blood samples, multiple frequency bioimpedance, 24‐h ambulatory blood pressure measurement, and 24‐h urine collection were obtained 1 week before banding and 1 and 2 weeks after. All measures were obtained on the same non‐dialysis weekday. Aldosterone and renin were measured with IDS‐iSYS chemiluminescence immunoassay, and angiotensin II by radioimmunoassay (Bidlingmaier et al., 2012; Marie Kappelgaard et al., 1976). To assess changes in BNP secretion, the more stable amino‐terminal NT‐proBNP was measured by Atellica chemiluminescence immunoassay (Hunt et al., 1995; IFCC,C‐CB). Mid‐regional proANP was quantitated on a Kryptor Plus platform (Thermo Fisher) (Hunter et al., 2011).

Body composition was measured using a multiple frequency bioimpedance (Fresenius Medical Body Composition Monitor), while 24‐h ambulatory blood pressure was assessed by a brachial cuff‐based ambulatory oscillometric device (Mobil‐O‐Graph®).

The patient received standard treatment for a high‐flow AVF and did not undergo any experimental interventions. Written informed consent for publication of the case report was obtained from the patient.

## 4 RESULTS

**FIGURE 1:**
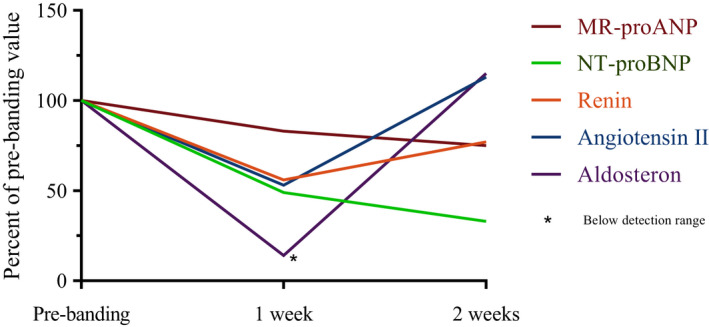
Hormone levels before and after arteriovenous fistula banding

Banding resulted in a decrease of AVF flow from >4 to 1.0 L/min. Clinical and paraclinical characteristics before banding and 1 and 2 weeks after banding are shown in Table 1. Banding resulted in a marked progressive suppression of NT‐proBNP and MR‐proANP while renin, angiotensin II, and aldosterone showed a transient decrease before returning to pre‐banding levels (Figure 1). Blood pressure increased and heart rate decreased. Sodium excretion and 24‐h diuresis increased, while body weight decreased, and the residual renal function determined by creatinine clearance tended to decrease (Table 1).

**TABLE 1:** Clinical and paraclinical characteristics before and after arteriovenous fistula banding

Characteristics	Pre‐banding	+1 week	+2 weeks
Weight (kg)	91.2	90.0	90.6
Body mass index (kg/m^2^)	33.5	33.1	33.3
Total body water (L)	32.5	32.6	34.5
ECW (L)^a^	16.5	16.5	17.3
ICW (L)^b^	16.0	16.1	17.2
ECW/ICW	1.0	1.0	1.0
Systolic blood pressure (mm Hg)^c^	153	156	157
Diastolic blood pressure (mm Hg)^c^	93	98	95
Heart rate (bpm^d^)^c^	71	69	66
Diuresis (ml/24 h)	1800	2000	2300
Creatinine clearance (ml/min)	5.6	4.0	5.0
Urinary sodium (mmol/24 h)	149	150	170
NT‐proBNP^e^ (pmol/L)	1440	702	470
MR‐proANP (pmol/L)^f^	860	712	647
Renin (×10^−3^ IU/L)	20.4	11.5	15.7
Angiotensin II (pmol/L)	4.7	2.5	5.3
Aldosterone (pmol/L)	731.4	<102.5^g^	843.2

^a^ Extracellular water.

^b^ Intracellular water.

^c^ Mean 24‐h ambulatory monitoring.

^d^ Beats per minute.

^e^ N‐terminal pro‐brain natriuretic peptide.

^f^ Mid‐regional pro‐atrial natriuretic peptide.

^g^ Below detection range.

## 5 DISCUSSION

This case report presents the short‐term physiological effects of sudden changes in AVF flow. The parameters that changed the most upon banding were the blood pressure and volume‐controlling hormones that decreased along with a reduction in heart rate, an indicator for reduced sympathetic activation.

Previous studies established an association between AVF flow and natriuretic peptides with an increase in ANP and BNP plasma concentrations after AVF creation and a decrease in BNP level associated with AVF closure (Iwashima et al., 2002; Jaques & Davenport, 2020; Rao et al., 2019). In accordance, we observed a decrease in ANP and BNP following AVF banding. ANP and BNP are released in response to increased ventricular pressure or stretch and ANP also in response to atrial stretch, and both are regarded as markers of cardiac strain (Iwashima et al., 2002; LaPointe, 2005; Yasue et al., 1994). The hemodynamic changes following AVF creation are known to induce left ventricular hypertrophy, ventricular and atrial dilatation, and have been linked to the development of congestive heart failure, which likely explains the observed decrease in natriuretic peptides following AVF flow reduction (Basile et al., 2007; Duque et al., 2015; MacRae et al., 2004). In line with this, closure of AVF in kidney transplant recipients and flow reduction in patients with high‐flow AVF improve heart structure and function (Rao et al., 2019; Unger et al., 2002; Valerianova et al., 2021). A cutoff value for a deleterious AVF flow, however, remains to be established in international guidelines (Lok et al., 2020).

A recent meta‐analysis found a significant blood pressure reduction following AVF creation in patients with end‐stage kidney disease and, conversely, an increase in blood pressure after AVF ligation (Scholz et al., 2019). Similarly, we observed an increase in total body water, extracellular fluid, and blood pressure following reduced AVF flow and natriuretic peptide concentrations. ANP and BNP are known to decrease blood pressure through RAAS inhibition, increased natriuresis, aquaresis, and vasodilation (Brunner‐La Rocca et al., 2001; Epstein et al., 1998; Loutzenhiser et al., 1988; Nishikimi et al., 2006). As expected, following banding we observed an increase in blood pressure, but also an unexpected temporary decrease in RAAS levels. A probable explanation is the sudden decrease in shunted blood flow following AVF banding, which causes an increase in effective circulating blood volume and blood pressure, likely suppressing RAAS and sympathetic nerve activity. This probably overrules any direct effects of ANP and BNP on RAAS. It is noteworthy that aldosterone was the RAAS component that showed the largest rebound when natriuretic peptide concentrations continued to decrease. This could reflect relief from direct suppression by natriuretic peptides of aldosterone release from glomerulosa cells.

Recently, observational studies found an association between AVF establishment and delayed kidney function decline in patients with end‐stage kidney disease (Bénard et al., 2019; Dupuis et al., 2021; Golper et al., 2015; Sumida et al., 2016). Several pathophysiological mechanisms explaining this association have been proposed. (Bénard et al., 2019; Golper et al., 2015). One suggestion is enhanced renal protection against ischemic injuries induced by subclinical arterial steal and hypoperfusion distal to the AVF––so‐called remote ischemic preconditioning (Zarbock et al., 2015). Other potential mechanisms include increased cardiac output and lowered systemic peripheral resistance and arterial stiffness, favoring renal perfusion (Korsheed et al., 2011, 2013; Ori et al., 1996). In this case report, we found that creatinine clearance paralleled the immediate decrease in ANP, BNP, and RAAS levels and tended to decrease 1‐week post‐banding. The abrupt decrease in natriuretic peptides and angiotensin II could account for the decreased creatinine clearance since they all support glomerular filtration by differential effects on the afferent and efferent arterioles (Hilgers & Mann, 1996; Jensen et al., 1999; Loutzenhiser et al., 1988; Nishikimi et al., 2006). There are existing pharmacological agents (i.e., neprilysin inhibitors) that increase the plasma concentrations of ANP and BNP (Vasquez et al., 2020). Use of these agents could be especially advantageous in patients with chronic kidney disease, which should be investigated in future studies.

The present study is a casuistic report and data should be confirmed in larger cohorts. Future studies are warranted to evaluate whether AVF establishment is associated with hormonal changes that could preserve remaining kidney function and improve fluid homeostasis in patients with end‐stage kidney disease. If a causal beneficial impact of AVF creation on kidney function is confirmed, early AVF creation in selected patients could potentially lead to delayed hemodialysis initiation and thereby reduced morbidity and mortality in patients with end‐stage kidney disease. Due to the risk of congestive cardiac failure in vulnerable patients, however, optimal timing of AVF creation and need for AVF banding likely needs to be tailored to the individual patient based on severity of chronic kidney disease and evaluation of co‐existing heart disease.

In summary, we found that reduction in AVF flow from >4 to 1 L/min was associated with increased total body water and blood pressure and a marked reduction in NT‐proBNP and MR‐proANP concentrations along with a transient decrease in RAAS and a small decrease in creatinine clearance. In conclusion, high‐flow AVF is associated with increased RAAS and natriuretic peptides that may support remaining kidney function.

## CONFLICT OF INTEREST

The authors declare that they have no conflict of interest.

## AUTHOR CONTRIBUTION

CLMO drafted the manuscript. CLMO, KL, BLJ, and DH contributed to the conception and design of the work. All took park in the analysis and interpretation of data, and all critically revised the manuscript and gave final approval.
